# Predictive significance of circulating tumor DNA against patients with T790M-positive EGFR-mutant NSCLC receiving osimertinib

**DOI:** 10.1038/s41598-023-48210-5

**Published:** 2023-11-27

**Authors:** Ou Yamaguchi, Norimitsu Kasahara, Hiroshi Soda, Hisao Imai, Ichiro Naruse, Hiroyuki Yamaguchi, Miki Itai, Kohei Taguchi, Megumi Uchida, Noriaki Sunaga, Toshitaka Maeno, Koichi Minato, Hiromi Tomono, Daiki Ogawara, Hiroshi Mukae, Yu Miura, Ayako Shiono, Atsuto Mouri, Hiroshi Kagamu, Kyoichi Kaira

**Affiliations:** 1 Present Address: Department of Respiratory Medicine, International Medical Center, Comprehensive Cancer Center, Saitama Medical University, 1397-1 Yamane, Hidaka-City, Saitama, 350-1298 Japan; 2 Innovative Medical Research Center, Gunma University Hospital, 3-39-15, Showa-machi, Maebashi, Gunma 371-8511 Japan; 3 Department of Respiratory Medicine, Sasebo City General Hospital, Nagasaki, Japan; 4 Division of Respiratory Medicine, Gunma Prefectural Cancer Center, Ota, Japan; 5 Department of Respiratory Medicine, Hidaka Hospital, Kagoshima, Japan; 6 Department of Respiratory Medicine, Nagasaki University Hospital, Nagasaki, Japan; 7 Department of Respiratory Medicine, Takasaki General Medical Center, Takasaki, Japan; 8 Department of Respiratory Medicine, Gunma University Graduate School of Medicine, Maebashi, Japan; 9 Department of Respiratory Medicine, Fukuoka Wajiro Hospital, Fukuoka, Japan

**Keywords:** Cancer, Oncology

## Abstract

Circulating tumor DNA (ctDNA) provides molecular information on tumor heterogeneity. The prognostic usefulness of ctDNA after first-line epidermal growth factor receptor (*EGFR*) tyrosine kinase inhibitors (TKIs) are limited. Therefore, the present study evaluated ctDNA during osimertinib administration as a second-line or more setting to identify the relationship between *EGFR* mutation levels and outcomes in patients with advanced non-small cell lung cancer (NSCLC). Forty patients with *EGFR* T790M-positive NSCLC receiving osimertinib after prior EGFR-TKI treatment were registered. Plasma samples were collected at osimertinib pretreatment, after 1 month of treatment, and at the time of progressive disease (PD). ctDNA analysis was performed by digital polymerase chain reaction. The detection rate of copy numbers of exon 19 deletion, L858R, and T790M in plasma samples was significantly lower 1 month after osimertinib than at pretreatment, and significantly higher at PD than at 1 month, whereas that of C797S was significantly higher at PD than at 1 month. No statistically significant difference was observed in the copy numbers of exon 19 deletion, L858R, T790M, and C797S between complete response or partial response and stable disease or PD. The detection of T790M at PD after osimertinib initiation was a significant independent prognostic factor for predicting shorter prognosis, and the presence of major *EGFR* mutations at pretreatment and PD was closely linked to worse survival after osimertinib initiation. Molecular testing based on ctDNA is helpful for predicting outcomes of osimertinib treatment in T790M-positive NSCLC after previous EGFR-TKI treatment.

## Introduction

Molecular targeting agents are useful to improve the prognosis of patients with cancer with driver mutations^1^. Among patients with advanced non-small cell lung cancer (NSCLC), epidermal growth factor receptor (*EGFR*) mutation is identified as the predominant target, and *EGFR* tyrosine kinase inhibitor (TKI) administration is the standard treatment for patients with NSCLC harboring *EGFR* mutation. In particular, osimertinib, as a third-generation EGFR-TKI, is administered as a first-line treatment^2^, but it can also be effective for patients with previously treated NSCLC harboring T790M-positive *EGFR* mutation, based on the AURA3 trial^3^. If T790M is detected in progressive disease (PD) after first- or second-generation EGFR-TKI administration, osimertinib is applicable to such patients. However, approximately 25% of patients resistant to first- or second-generation EGFR-TKIs can receive osimertinib because of T790M-positive testing^4^. As the presence of T790M within tumor specimens cannot predict the outcome of osimertinib administration, the development of an useful predictor is required to distinguish responders from non-responders.

Recently, Ariyasu et al.^5^ evaluated the ratio of T790M to EGFR-activating mutations in cytological samples from 33 patients with NSCLC receiving osimertinib using droplet digital polymerase chain reaction (PCR) and found a significant correlation between the T790M ratio and the tumor reduction rate. In addition, the clearing of all EGFR mutations from the blood after osimertinib initiation significantly predicted its efficacy and outcome in 82 pretreated patients receiving osimertinib, and all clearing of T790M and sensitizing mutations is necessary for a positive predictor of osimertinib^6^.

The assessment of circulating tumor DNA (ctDNA) is a noninvasive strategy for cancer diagnosis; in particular, plasma ctDNA testing to examine sensitizing *EGFR* and T790M mutations was approved in 2015^7^. *EGFR* and T790M plasma testing was evaluated using several techniques, such as PCR and next-generation sequencing. Although liquid biopsy is more feasible than tissue biopsy, there are some doubts about the sensitivity of its detection. Tumor tissue genotyping exhibits a high sensitivity and specificity for molecular analysis, but, yields a limitation about tumor availability and accessibility. The turnaround time for genetic analysis is longer than that for liquid biopsy. Thus, liquid biopsy could overcome these limitations because of faster turnaround time and less invasive procedure^8^. Digital PCR is a recently developed method. It has higher detection sensitivity than conventional PCR and has recently attracted attention for the detection of ctDNA^9,10^. However, a recent report analyzed extracted ctDNA using non-digital platforms (the cobas EGFR Mutation Test) and digital platforms (BEAMing dPCR), and demonstrated that both platforms yielded a high sensitivity for T790M mutation detection^11^. Non-digital platform by the cobas EGFR Mutation Test is also considered as comparable technology to digital PCR. In several studies, the ratio of T790M to *EGFR*-activating mutations in ctDNA, plasma pretreatment T790M level, and fraction of *EGFR*-mutant ctDNA in plasma have been reported to be potential markers of prognosis in patients receiving osimertinib^12–14^. However, there are no established markers to predict the outcome of osimertinib treatment in patients with T790M-positive *EGFR* mutation.

Based on this background, we conducted a prospective study to investigate the predictive markers of osimertinib in patients with T790M-positive *EGFR*-mutant NSCLC using digital PCR.

## Results

### Patient demographics

Patient demographics are shown in Table 1. Twenty-four patients had exon 19 deletion, 15 harbored L858R, and one patient presented with L861Q, as detected using the cobas method. Forty blood samples were collected from patients before osimertinib initiation, 36 blood samples were collected from patients 1 month after osimertinib administration, and 20 blood samples were collected from patients with PD after osimertinib treatment. Nine of the patients remained on osimertinib treatment without progression at the time of data cutoff, whereas 31 patients experienced PD. The median age was 69 (range, 33–85) years, and 28 (70.0%) patients were women. Thirty-one (77.6%) patients were never-smoker, 35 (87.5%) had a performance status (PS) score of 0–1, and all patients were classified as having adenocarcinoma histology. Thirty-four (85%) patients had stage III or IV disease, and 6 (15%) patients had postoperative recurrence.

**Table 1:** Patient’s demographics.

Different variables		N = 40 (%)
Age (year)	Median (range)	69 (33–85)
Gender	Male/Female	12/28 (30.0/70.0)
Clinical stage	III/IV/Ope rec.	4/30/6 (10.0/75.0/15.0)
Smoking history	Yes/No	9/31 (22.4/77.6)
ECOG PS	0–1/2	35/5 (87.5/12.5)
Histology	AC/other	40/0 (100/0)
EGFR mutation status	Exon 19/L858R/L861Q	24/15/1 (60.0/37.5/2.5)
Numbers of prior treatment	1/2 or more lines	29/11 (72.5/27.5)
Prior EGFR-TKIs regimens	Gefitinib/Erlotinib/Afatinib	11/12/17 (27.5/30.0/42.5)
Brain metastases	Yes/No	17/23 (42.5/57.5)
PD-L1 expression	≥ 50%/< 50%/unknown	1/17/22 (2.5/42.5/55.0)

ECOG, eastern cooperative oncology group; PS, performance status; PD-L1, programmed death ligand-1; AC, adenocarcinoma; EGFR, epidermal growth factor receptor; TKIs, tyrosine kinase inhibitors; Ope rec, recurrence after operation.

### EGFR mutation assessment in plasma circulating tumor DNA (ctDNA) samples

**Figure 1:**
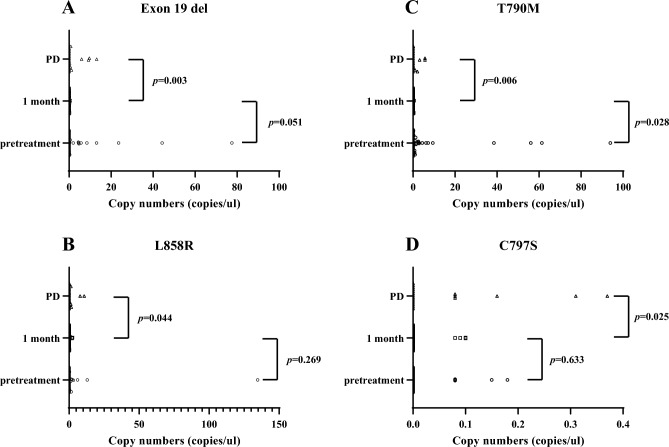
Distribution of epidermal growth factor receptor (*EGFR*) mutant-allele copy number in plasma samples before osimertinib (pretreatment), 1 month after its administration, and at progressive disease to its treatment, according to the *EGFR* mutation status of exon 19 deletion (**A**), exon 21 L858R (**B**), T790M (**C**), and C797S (**D**).

**Figure 2:**
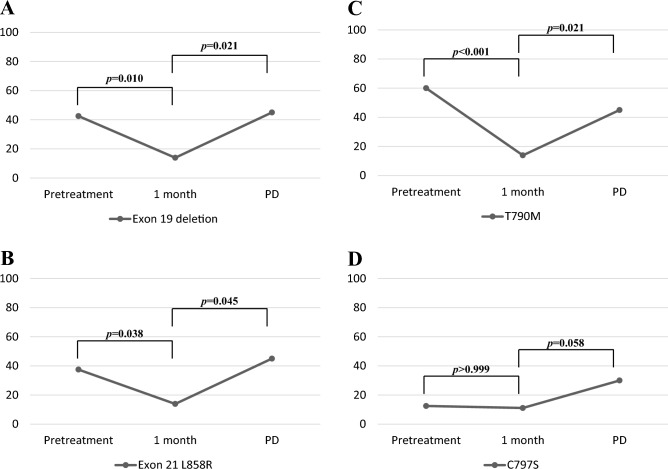
Comparison of positive rate of copy numbers in exon 19 deletion (**A**), L858R (**B**), T790M (**C**), and C797S (**D**), according to pretreatment, 1 month, and at progressive disease after osimertinib initiation.

In a total of 40 patients, the detection rates for *EGFR* sensitizing mutations at baseline, 1 month and PD after osimertinib were 80.0% 27.8% (32/40), (10/36), and 85.0% (17/20), respectively. In the exon 19 deletion assay, the copy numbers detected in plasma samples at pretreatment, at 1 month, and at PD after osimertinib treatment ranged from 0 to 77.5 (positive rate, 42.5% [17/40]), from 0 to 0.59 (positive rate, 13.9% [5/36]), and from 0 to 13.15 copies/µL (positive rate, 45.0% [9/20]), respectively, demonstrating a significant difference between 1 month and PD (*p* = 0.003) (Fig. 1A). In the L858R assay, the copy numbers in the plasma sample at pretreatment, at 1 month, and at PD after treatment ranged from 0 to 134.7 (positive rate, 37.5% [15/40]), from 0 to 2.41 (positive rate, 13.9% [5/36]), and from 0 to 10.83 copies/µL (positive rate, 40.0% [8/20]), respectively, indicating a significant difference between 1 month and PD (*p* = 0.044) (Fig. 1B). The copy numbers detecting T790M ranged from 0 to 93.9 at pretreatment (positive rate, 60.0% [24/40]), from 0 to 0.45 at 1 month (positive rate, 13.9% [5/36]), and from 0 to 5.67 copies/µL (positive rate, 45.0% [9/20]). There was a significant difference in the copy numbers between pretreatment and 1 month (*p* = 0.028) and between 1 month and PD (*p* = 0.006) (Fig. 1C). The copy numbers of C797S ranged from 0 to 0.18 at pretreatment (positive rate, 12.5% [5/40]), from 0 to 0.09 at 1 month (positive rate, 11.1% [4/36]), and from 0 to 0.31 at PD (positive rate, 30.0% [6/20]), and those at PD were significantly higher than those at 1 month (*p* = 0.025) (Fig. 1D). Moreover, the positive rate of copy numbers of exon 19 deletion, L858R, and T790M in plasma samples were significantly lower at 1 month after osimertinib treatment than at pretreatment and PD, whereas that of C797S was significantly higher at PD than at 1 month (Fig. 2).

### Association between plasma ctDNA and efficacy to osimertinib

Of the 40 patients, one achieved a complete response (CR), 24 had a partial response (PR), 11 had stable disease (SD), 3 displayed PD, and one had a non-evaluable lesion. The overall response rate (ORR) and disease control rate were 64.1% (25/39) and 92.3% (36/39), respectively. A comparison of the copy numbers based on the exon 19 deletion, L858R, T790M, and C797S between CR or PR (responders) and SD or PD (non-responders) was performed. However, there was no statistically significant difference in the copy numbers between them (Fig. A, online only). In addition, the positive rate of copy number did not differ significantly between responders and non-responders, except for exon 19 deletion at pretreatment (Fig. B, online only).

### Survival analysis according to plasma ctDNA

**Figure 3:**
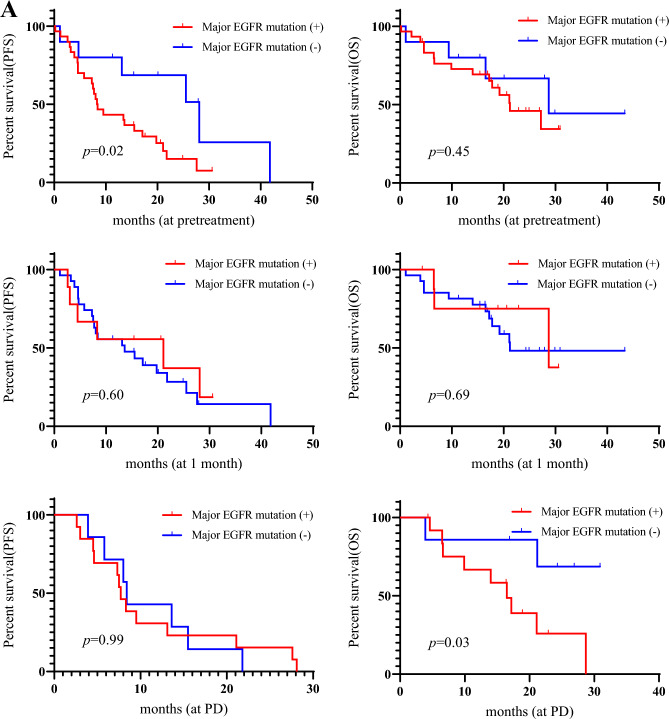

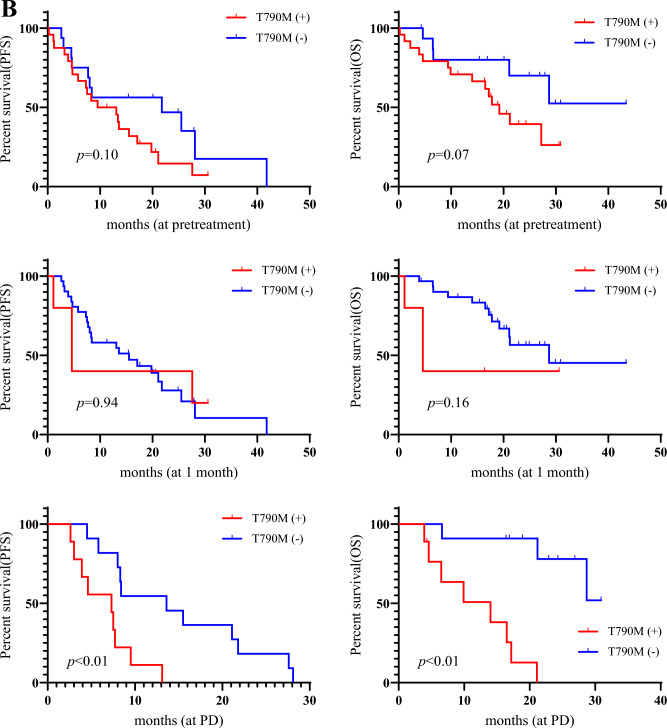
Kaplan–Meier curve according to major epidermal growth factor receptor (*EGFR*) mutation (**A**) and T790M (**B**) at pretreatment, 1 month, and progressive disease (PD) after osimertinib administration. Patients with detection of major *EGFR* mutation at pretreatment exhibited a significantly shorter progression-free survival (PFS) than those without it; moreover, there was significantly worse overall survival (OS) in patients with detection of major EGFR mutation at PD than those without it (**A**). Patients with T790M detection at PD yielded significantly worse PFS and OS than those without it (**B**).

The median PFS and OS were 13.1 months and 27.2 months, respectively. Twenty-five (62.5%) of the 40 patients experienced progression, and 19 (47.5%) patients died due to disease progression. Univariate and multivariate analyses were performed in all patients (Tables 2 and 3). Univariate analysis identified the detection of major *EGFR* mutation (exon 19 deletion and L858R) at pretreatment and detection of T790M at PD after osimertinib treatment for PFS (Table 2), and PS, brain metastasis, detection of major *EGFR* mutation at PD, and detection of T790M at PD after its treatment were considered significant predictors for OS (Table 3). Multivariate analysis confirmed that detection of major *EGFR* mutation at pretreatment and detection of T790M at PD were independent prognostic factors for predicting favorable PFS (Table 2), and detection of T790M at PD was identified as an independent factor for predicting worse OS (Table 3). Kaplan–Meier curves for PFS and OS according to the detection of major *EGFR* mutation and T790M are shown in Fig. 3A,B.

**Table 2:** Univariate and multivariate analysis in progression-free survival.

Different variables		Univariate analysis		Multivariate analysis	
		HR (95% CI)	*p*-value	HR (95% CI)	*p*-value
Age	< 65/≥ 65	1.10 (0.46–2.50)	0.81		
Gender	Male/Female	0.96 (0.37–2.25)	0.94		
Smoking history	Ever/Never	0.63 (0.18–1.67)	0.37		
ECOG PS	0–1/2	0.23 (0.05–1.61)	0.12		
EGFR mutation	Ex19 del/L858R	0.94 (0.40–2.30)	0.90		
Line of previous treatment	2/≥ 3	0.46 (0.18–1.18)	0.10		
Brain metastasis	Yes/No	1.51 (0.66–3.41)	0.31		
Detection of major mt (pre)	Yes/No	2.81 (1.14–8.52)	**0.02**	7.17 (1.37–13.2)	**0.01**
Detection of major mt at 1M	Yes/No	0.78 (0.28–1.87)	0.60		
Detection of major mt at PD	Yes/No	0.99 (0.38–2.71)	0.99		
Detection of T790M (pre)	Yes/No	1.86 (0.88–4.22)	0.10		
Detection of T790M at 1M	Yes/No	1.04 (0.29–2.82)	0.94		
Detection of T790M at PD	Yes/No	4.45 (1.49–14.86)	**< 0.01**	6.17 (1.94–22.5)	**< 0.01**
Detection of C797S (pre)	Yes/No	1.46 (0.42–3.87)	0.50		
Detection of C767S at 1M	Yes/No	1.86 (0.53–4.03)	0.29		
Detection of C797S at PD	Yes/No	1.53 (0.52–4.03)	0.41		
Decrease of plasma major mt	Yes/No	2.32 (0.97–6.45)	0.05		
Decrease of plasma T790M	Yes/No	1.65 (0.75–3.83)	0.21		

ECOG, eastern cooperative oncology group; PS, performance status; EGFR, epidermal growth factor receptor; major mt, major EGFR mutation (exon 19 deletion or L858R); (pre), at pretreatment; 1M, 1 month after osimertinib administration; PD, progressive disease after osimertinib administration; HR, hazard ratio; 95% CI, 95% confidence interval; Decrease of plasma major mt; decrease of major EGFR mutation in plasma samples from pretreatment to 1 month after osimertinib administration; Decrease of plasma T790M, decrease of T790M in plasma samples from pretreatment to 1 month after osimertinib administration.

Statistically significance values are in bold.

**Table 3:** Univariate and multivariate analysis in overall survival.

Different variables		Univariate analysis		Multivariate analysis	
		HR (95% CI)	*p*-value	HR (95% CI)	*p*-value
Age	< 65/≥ 65	0.44 (0.12–1.21)	0.11		
Gender	Male/Female	0.52 (0.15–1.45)	0.23		
Smoking history	Ever/Never	0.55 (0.12–1.68)	0.32		
ECOG PS	0–1/2	0.07 (0.20–0.28)	**< 0.01**	0.68 (0.12–5.19)	0.68
EGFR mutation	Ex19 del/L858R	0.68 (0.26–1.83)	0.44		
Line of previous treatment	2/ ≥ 3	0.39 (0.15–1.03)	0.05		
Brain metastasis	Yes/No	2.93 (1.14–7.87)	0.02	2.45 (0.48–19.2)	0.29
Detection of major mt (pre)	Yes/No	1.51 (0.54–5.37)	0.45		
Detection of major mt at 1M	Yes/No	0.77 (0.17–2.45)	0.69		
Detection of major mt at PD	Yes/No	4.50 (1.11–30.36)	**0.03**	0.61 (0.03–7.91)	0.71
Detection of T790M (pre)	Yes/No	2.39 (0.90–7.48)	0.07		
Detection of T790M at 1M	Yes/No	2.72 (0.61–8.79)	0.16		
Detection of T790M at PD	Yes/No	21.7 (3.81–409.2)	**< 0.01**	22.4 (2.50–672)	**< 0.01**
Detection of C797S (pre)	Yes/No	1.34 (0.31–4.09)	0.64		
Detection of C767S at 1M	Yes/No	2.47 (0.37–9.81)	0.29		
Detection of C797S at PD	Yes/No	0.88 (0.19–3.07)	0.85		
Decrease of plasma major mt	Yes/No	0.94 (0.33–3.06)	0.92		
Decrease of plasma T790M	Yes/No	1.86 (0.65–6.03)	0.24		

ECOG, eastern cooperative oncology group; PS, performance status; EGFR, epidermal growth factor receptor; major mt, major EGFR mutation (exon 19 deletion or L858R); (pre), at pretreatment; 1M, 1 month after osimertinib administration; PD, progressive disease after osimertinib administration; HR, hazard ratio; 95% CI, 95% confidence interval; Decrease of plasma major mt; decrease of major EGFR mutation in plasma samples from pretreatment to 1 month after osimertinib administration; Decrease of plasma T790M, decrease of T790M in plasma samples from pretreatment to 1 month after osimertinib administration.

Statistically significance values are in bold.

### Clinical efficacy on the ratio of T790M to major EGFR mutation

The clinical significance of the T790M/major *EGFR* mutation ratio at pretreatment was investigated, with a mean of 0.305 for CR/PR/SD and 0.185 for PD, without a significant difference (*p* = 0.632) (Fig. C1, online only). According to ROC analysis, the best cut-off value of the T790M/major *EGFR* mutation ratio was 0.727. No statistically significant difference in PFS and OS was observed between patients with T790M/major *EGFR* mutation ≥ 0.727 and < 0.727 (Fig.  C2,C3, online only).

## Discussion

This was a prospective observational study evaluating the prognostic significance of ctDNA, such as exon 19 deletion, L858R, T790M, and C797S *EGFR* mutations, in patients with previously treated NSCLC harboring T790M *EGFR* mutation who received osimertinib. The detection of ctDNA in plasma samples by digital PCR exactly reflected the change in exon 19 deletion, L858R, and T790M at 1 month and PD after osimertinib administration. We found that the detection of T790M at PD after osimertinib initiation was a significant independent prognostic factor for predicting worse OS, and that the detection of major *EGFR* mutations at pretreatment and T790M at PD also affected the outcome after osimertinib initiation. Currently, osimertinib is clarified as a first-line EGFR-TKI; thus, the frequency of its administration as a second-line or more lines is decreasing. However, the detection of blood samples using digital PCR is a noninvasive technique that is convenient for daily practice, and an improvement in the detection rate is warranted to realize the development of an optimal predictor.

The analysis of *EGFR* mutation in tissue and plasma from the AURA3 trial reported that the detection of plasma T790M was related to a larger baseline tumor size, and PFS after osimertinib (median, 12.5 vs. 8.3 months) was longer in patients with a cobas plasma T790M-negative status at pretreatment compared to those with a plasma T790M-positive status^15^. PFS in patients with NSCLC harboring T790M who received osimertinib tended to be worse in patients with high T790M copy number at pretreatment, as assessed by cell-free plasma DNA using digital, rather than those with low T790M copy number. Moreover, no significant difference in ORR was recognized according to T790M copy numbers^16^. Although another study also assessed the association between the utility of plasma T790M mutant copy number at pretreatment and the prognostic role of osimertinib using digital PCR, a high plasma T790M copy number was associated with worse PFS and OS compared to a low T790M copy number^17^. In addition, a high copy number of active *EGFR* mutations in plasma samples has been reported to be closely associated with poor outcomes after osimertinib treatment^13^. The results of these studies indicated that a high copy number of plasma T790M or activating *EGFR* mutations was a useful marker for predicting worse PFS in patients with NSCLC harboring positive *EGFR* T790M who were treated with osimertinib^13,15–17^. Ding et al.^12^ reported that high plasma T790M level at pretreatment was related to superior disease control in patients with NSCLC with advanced *EGFR* T790M treated with osimertinib. In our study, the detection of major plasma *EGFR* mutation (exon 19 deletion or L858R) at pretreatment could predict shorter PFS after osimertinib administration, but not plasma T790M copy number. Although previous studies have focused on the association between plasma *EGFR* mutation copy numbers and the efficacy of osimertinib at pretreatment, Sakai et al.^18^ evaluated the clinical significance of monitoring the ctDNA of EGFR-TKI-sensitizing mutations and *EGFR* T790M mutation in *EGFR* T790M-positive patients with NSCLC at pretreatment, on day 1 of treatment cycle 4 or 9, and at the diagnosis of PD using digital PCR. In their study, rebound of sensitizing *EGFR* mutation and T790M was observed at PD, and ctDNA monitoring for sensitizing *EGFR* mutation at four cycles was better for predicting the outcome after osimertinib^18^. We found that the detection rate of ctDNA by digital PCR significantly decreased 1 month after osimertinib initiation, and that the detection of plasma T790M at PD was closely associated with worse outcomes. Although ctDNA monitoring in the early phase after osimertinib may not be useful for prognostic prediction, the detection of major *EGFR* mutation at pretreatment is predictive of PFS after osimertinib based on previous studies.

Several studies have investigated the prognostic significance of the ratio of T790M to major *EGFR* mutation at baseline in patients with advanced *EGFR* T790M-positive NSCLC receiving osimertinib and reported that a higher ratio is closely related to tumor shrinkage and favorable survival^13,19^. Two researchers observed that the ratio was significantly higher in patients with CR/PR/SD than in those with PD^13,19^. In the present study, the ratio of T790M/major *EGFR* mutation at pretreatment could not predict the outcome or tumor shrinkage after osimertinib administration. A previous study confirmed that the amount of plasma T790M at pretreatment is not a reliable biomarker for tumor response and survival^13,19^. This finding is similar to the results of the present study. Although the absence of major *EGFR* mutation in plasma at pretreatment may be a significant surrogate marker for the outcome after osimertinib treatment, we believe that baseline T790M level is not closely associated with response and prognostic prediction.

Our study has some limitations. First, the sample size was small, which may have biased the results. Second, the current study focused on patients harboring the T790M EGFR mutation after first- or second-generation EGFR-TKI administration. Currently, osimertinib is frequently administered to patients with naive EGFR-TKIs. Therefore, our digital PCR technique should be examined as an exploratory investigation for a predictive marker of first-line osimertinib. Moreover, we defined a copy number of 0 (copies/µL) as the cut-off value for further investigation. The cut-off values of copy number are different according to individual studies; therefore, it is uncertain whether an optimal cut-off value is determined by digital PCR. Finally, we were unable to investigate the mechanisms of resistance to osimertinib, except for C797S. Resistance mechanisms, such as the bypass signaling pathway, PTEN loss, MET amplification, MYC amplification, and small cell lung carcinoma transformation, have been described in previous reports^20–22^. Since resistance to osimertinib is closely associated with many tumor mutations, it is difficult to identify specific markers.

In conclusion, the detection of plasma T790M at PD after osimertinib treatment is identified as a significant predictor of worse outcomes after osimertinib administration. The detection of major *EGFR* mutations during pretreatment and PD also affects the outcome after treatment. Further investigation using a large-scale sample is warranted to confirm the results of our study.

## Methods

### Patients

A total of 40 patients with advanced *EGFR* T790M-mutant NSCLC on PD after first- or second-generation EGFR-TKI administration were registered in a prospective, observational multicenter study from August 2016 to December 2019. The inclusion criteria in this study were as follows: age ≥ 20 years, cytologically or histologically confirmed NSCLC harboring *EGFR* mutations, PD after first- or second-generation EGFR-TKI (gefitinib, erlotinib, or afatinib) administration, and verified EGFR T790M mutation in liquid and/or tissue re-biopsy. Clinical data were extracted from the medical records. This study was conducted according to the international guidelines on Good Clinical Practice and the Declaration of Helsinki and was approved by the institutional ethics committee (Gunma University Hospital Clinical Research Review Board) on 4th of April, 2017. (Registration number: 1524) Written informed consent was obtained from all participating individuals.

### Treatment and evaluation

All patients were treated with osimertinib 80 mg daily as a starting dose, with dose reductions or interruptions based on the clinician’s discretion because of PD or therapeutic toxicity. Physical examination, complete blood count, biochemical testing, and adverse events were measured according to the judgment of each chief physician. Any toxicity was graded based on the Common Terminology Criteria for Adverse Events version 4.0. Tumor response was examined according to the Response Evaluation Criteria in Solid Tumors version 1.1^23^.

### Blood sample collection and epidermal growth factor receptor (EGFR) mutation analysis

Peripheral blood samples (20 mL) were collected in ethylenediaminetetraacetic acid tubes before pretreatment, 1 month after osimertinib treatment, and at PD. The samples were centrifuged at 3000 rpm for 15 min at room temperature and stored at − 80 °C until analysis. ctDNA was isolated using a cfDNA Sample Preparation Kit (Roche Molecular Systems).

EGFR mutation testing using Cobas was performed according to the manufacturer’s protocol. Mutation analysis was performed by PCR using a Cobas z480 analyzer (Roche Molecular Systems).

Droplet digital PCR (Bio-Rad) was performed according to previously reported techniques^8^, and sensitizing EGFR mutations, EGFR T790M, and EGFR C797S were detected. The primers and probe for the detection of sensitizing EGFR mutations and EGFR T790M were provided by Bio-Rad using the QUANTSOFT analytical software package (Bio-Rad). Primers and probes for detecting EGFR C797S were obtained from Riken Genetics. In the present study, we did not analyze the detection of L861Q based in the ddPCR.

### Statistical analyses

For statistical analyses, we used Student’s *t-test* and the χ^2^ test for continuous and categorical variables, respectively. The statistical significance level was set at *p* < 0.05. Progression-free survival (PFS) was defined as the time from the day of initial osimertinib treatment to either earlier that of disease progression or death. Overall survival (OS) was defined as the time from the day of initial osimertinib treatment to that of death from any cause. The Kaplan–Meier method was used to estimate survival as a function of time, and survival differences were analyzed using the log-rank test. Univariate and multivariate analyses according to different variables were performed using logistic regression analysis. In this study, a copy number of 0 (copies/µL) was defined as negative, and a copy number greater than 0 was defined as positive, according to previous investigation^24^. The predictive value of T790M/major *EGFR* mutation ratio at pretreatment was determined by receiver operating characteristic (ROC) curve analyses, which were used for PFS to identify an optimal cut-off value. All statistical analyses were performed using GraphPad Prism (version 8.0; GraphPad Software, San Diego, CA, USA) and JMP 14.0 (SAS Institute Inc., Cary, North Carolina, USA).

### Supplementary Information

Supplementary Figures.

Supplementary Legends.

## Data Availability

The datasets used and/or analysed during the current study available from the corresponding author on reasonable request.
