# Supplementary material for: Predictive significance of circulating tumor DNA against patients with T790M-positive EGFR-mutant NSCLC receiving osimertinib
Source: Sci Rep. 2023 Nov 27;13:20848. doi: 10.1038/s41598-023-48210-5 (PMC10682450; doi:10.1038/s41598-023-48210-5)
Supplement: Supplementary file 1 — Supplementary Figures. [file 41598_2023_48210_MOESM1_ESM.pptx]

## Slide 1
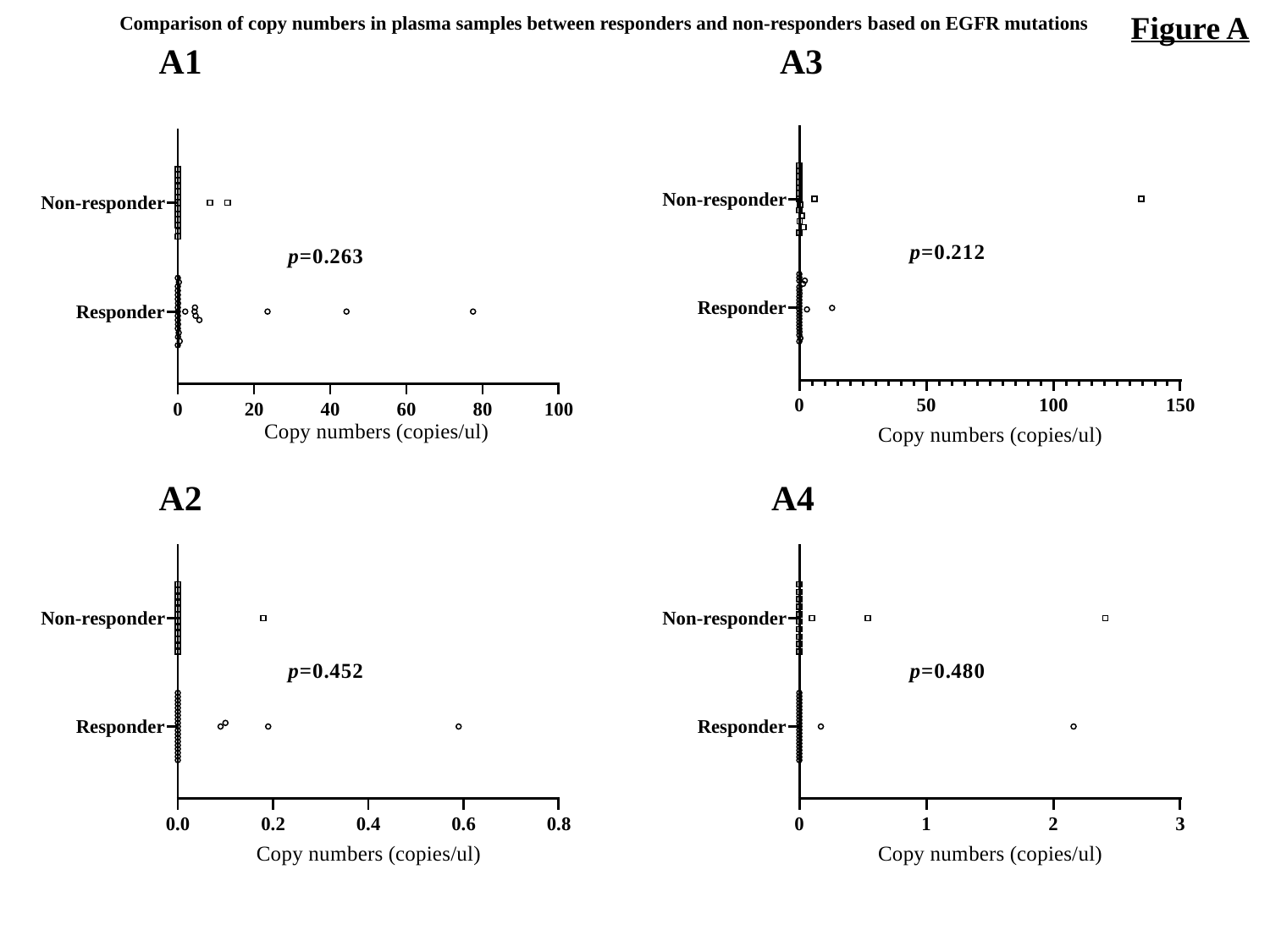

Figure A
Comparison of copy numbers in plasma samples between responders and non-responders based on EGFR mutations
A1
A3
A2
A4

## Slide 2
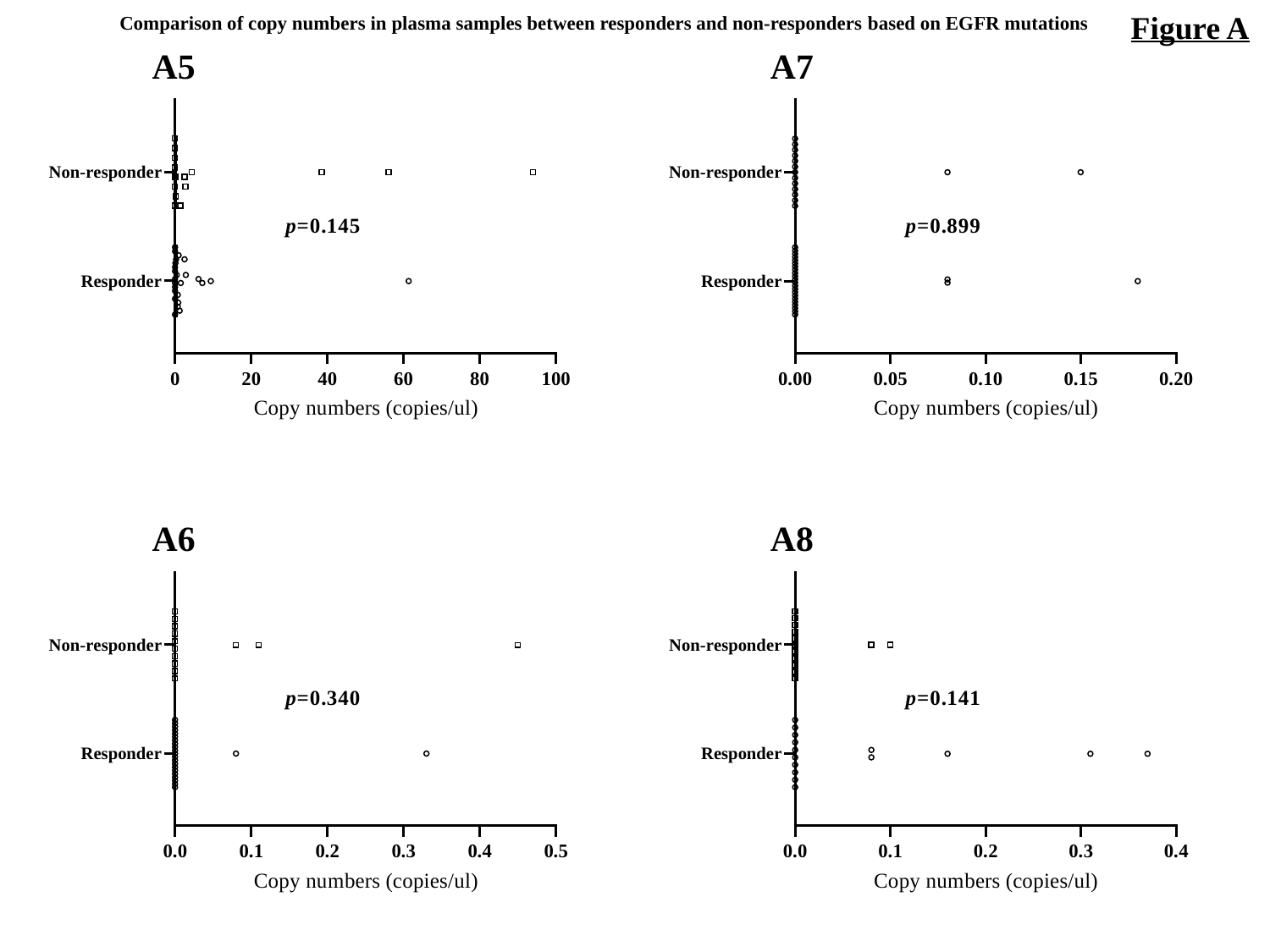

Figure A
Comparison of copy numbers in plasma samples between responders and non-responders based on EGFR mutations
A5
A7
A6
A8

## Slide 3
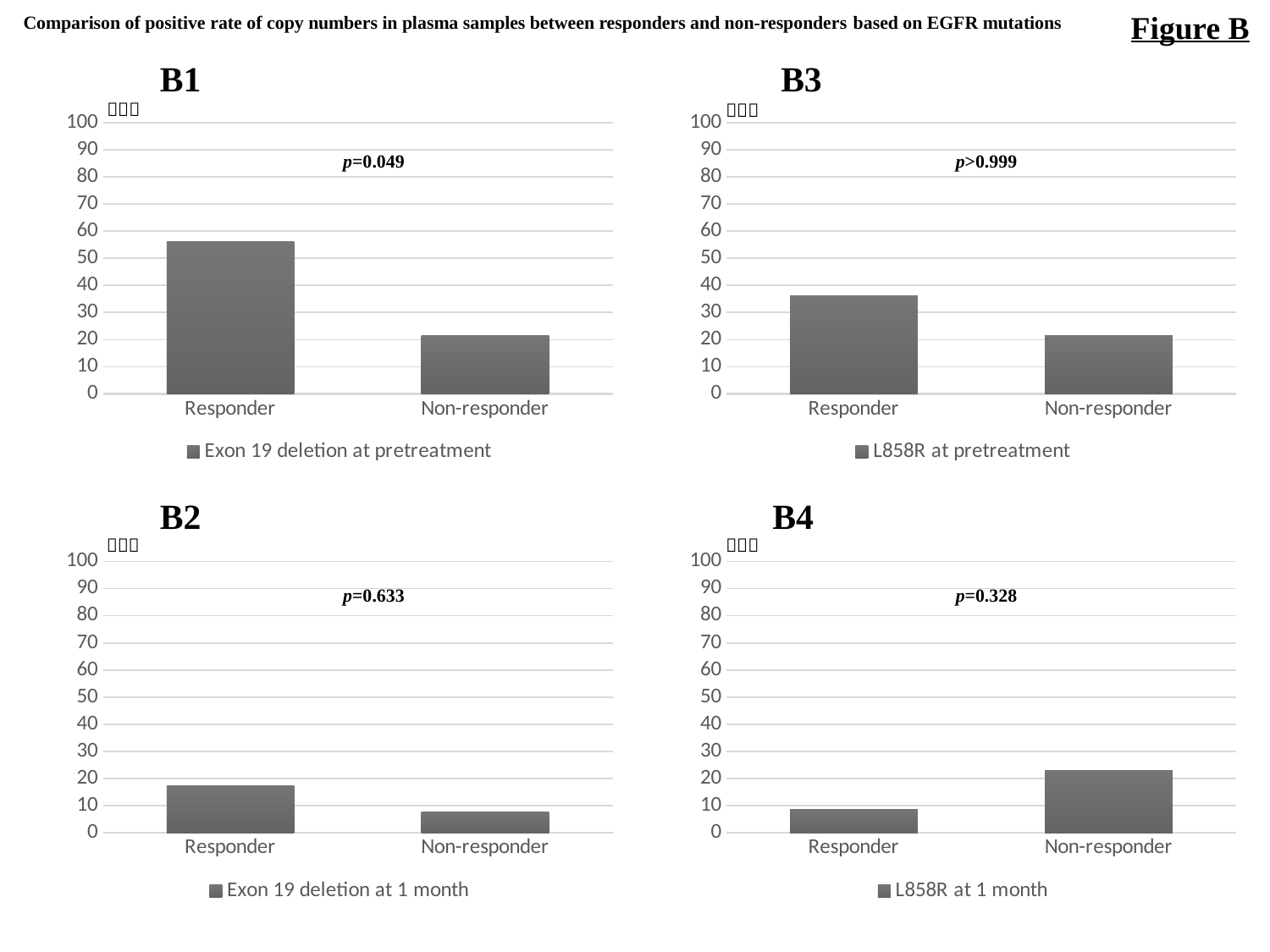

Figure B
Comparison of positive rate of copy numbers in plasma samples between responders and non-responders based on EGFR mutations
B1
B3
（％）
（％）
### Chart
| Category | L858R at pretreatment |
|---|---|
| Responder | 36.0 |
| Non-responder | 21.4 |
### Chart
| Category | Exon 19 deletion at pretreatment |
|---|---|
| Responder | 56.0 |
| Non-responder | 21.4 |p=0.049
p>0.999
B2
B4
（％）
（％）
### Chart
| Category | L858R at 1 month |
|---|---|
| Responder | 8.6 |
| Non-responder | 23.1 |
### Chart
| Category | Exon 19 deletion at 1 month |
|---|---|
| Responder | 17.3 |
| Non-responder | 7.6 |p=0.328
p=0.633

## Slide 4
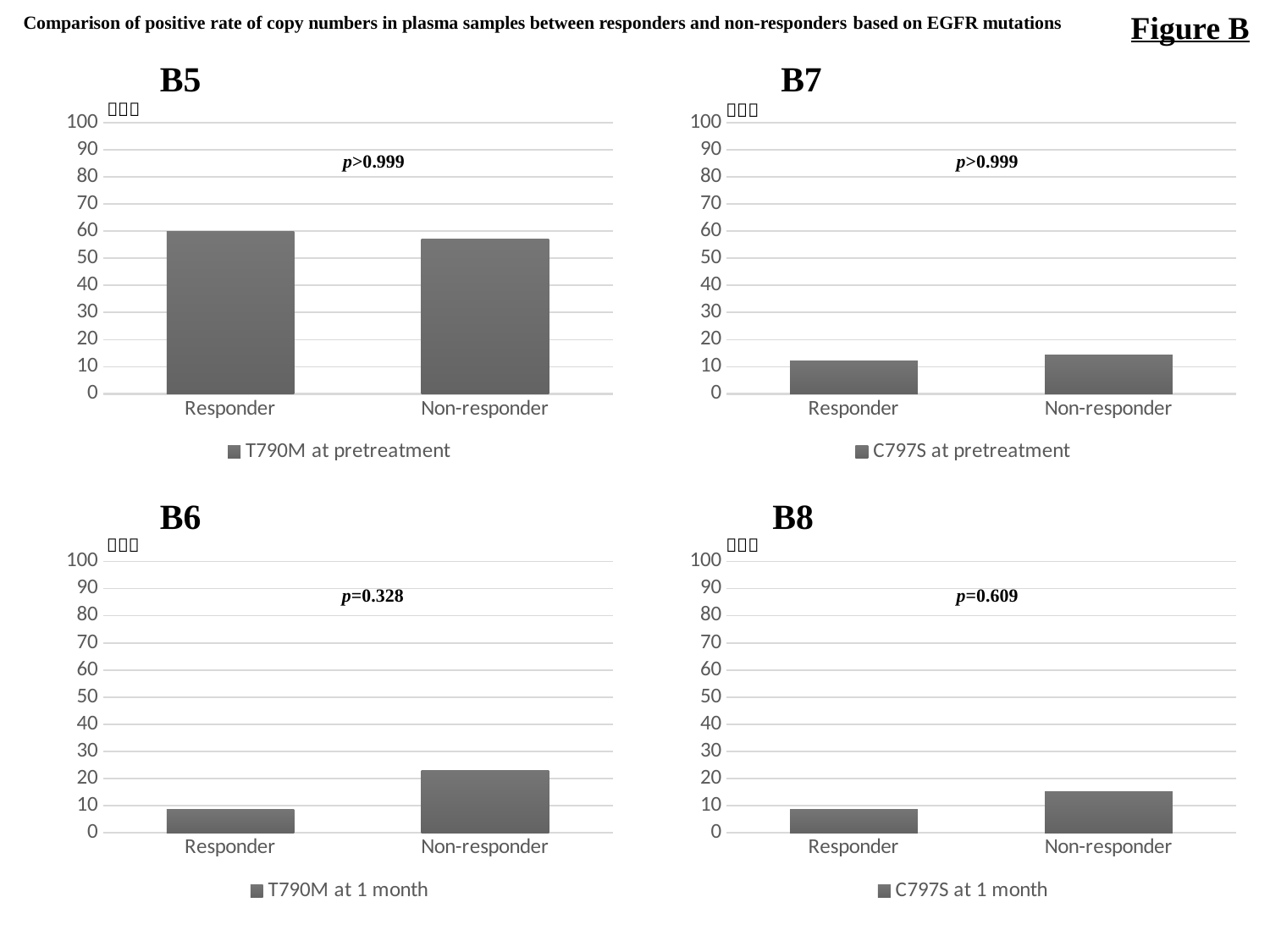

Figure B
Comparison of positive rate of copy numbers in plasma samples between responders and non-responders based on EGFR mutations
B5
B7
（％）
（％）
### Chart
| Category | C797S at pretreatment |
|---|---|
| Responder | 12.0 |
| Non-responder | 14.2 |
### Chart
| Category | T790M at pretreatment |
|---|---|
| Responder | 60.0 |
| Non-responder | 57.1 |p>0.999
p>0.999
B6
B8
（％）
（％）
### Chart
| Category | C797S at 1 month |
|---|---|
| Responder | 8.6 |
| Non-responder | 15.3 |
### Chart
| Category | T790M at 1 month |
|---|---|
| Responder | 8.6 |
| Non-responder | 23.1 |p=0.328
p=0.609

## Slide 5
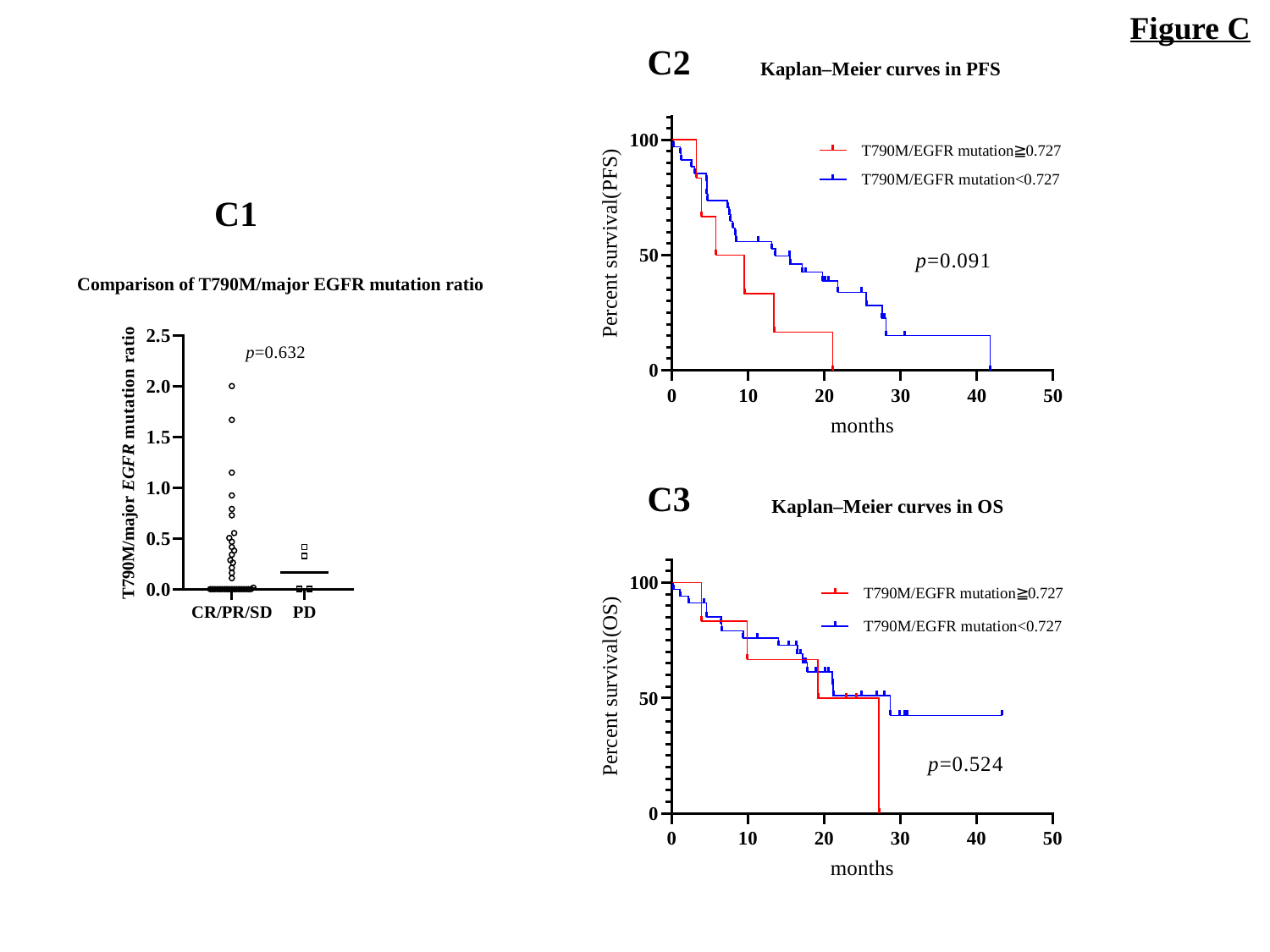

Figure C
C2
Kaplan–Meier curves in PFS
C1
Comparison of T790M/major EGFR mutation ratio
C3
Kaplan–Meier curves in OS
