# Supplementary material for: Predictive significance of circulating tumor DNA against patients with T790M-positive EGFR-mutant NSCLC receiving osimertinib
Source: Sci Rep. 2023 Nov 27;13:20848. doi: 10.1038/s41598-023-48210-5 (PMC10682450; doi:10.1038/s41598-023-48210-5)
Supplement: Supplementary file 2 — Supplementary Legends. [file 41598_2023_48210_MOESM2_ESM.docx]

**Supplemental figures**

**Figure A**

Comparison of copy numbers in plasma samples between responders (complete response or partial response) and non-responders (stable disease or progressive disease) based on different epidermal growth factor receptor mutations.

No statistically significant difference in copy numbers between responders and non-responders was observed at pretreatment (**A1**) and 1 month after osimertinib (**A2**) administration in exon 19 deletion, at pretreatment (**A3**) and 1 month (**A4**) in L858R, at pretreatment (**A5**) and 1 month (**A6**) in T790M, and at pretreatment (**A7**) and 1 month (**A8**) in C797S.

**Figure B**

Comparison of positive rate of copy numbers in plasma samples between responders (complete response or partial response) and non-responders (stable disease or progressive disease) based on different epidermal growth factor receptor mutations.

There was a statistically significant difference in the positive rate between responders and non-responders at pretreatment for exon 19 deletion (B1); however, no statistically significant difference was observed at 1 month after osimertinib (**B2**) administration in exon 19 deletion, at pretreatment (**B3**) and 1 month (**B4**) in L858R, at pretreatment (**B5**) and 1 month (**B6**) in T790M, and at pretreatment (**B7**) and 1 month (**B8**) in C797S.

**Figure C**

Comparison of T790M/major epidermal growth factor receptor (EGFR) mutation ratio in patients with complete response/partial response/stable disease vs. progressive disease (**C1**). Kaplan–Meier curves in progression-free survival (**C2**) and overall survival (**C3**) based on the cut-off value (0.727) of T790M/major EGFR mutation calculated by receiver operating characteristic curve analysis.
